# Time Trends of the Outcomes and Treatment Options for Disseminated Intravascular Coagulation: A Nationwide Observational Study in Japan

**DOI:** 10.31662/jmaj.2020-0013

**Published:** 2020-09-23

**Authors:** Kazuma Yamakawa, Hiroyuki Ohbe, Kohei Taniguchi, Hiroki Matsui, Kiyohide Fushimi, Hideo Yasunaga

**Affiliations:** 1 Division of Trauma and Surgical Critical Care, Osaka General Medical Center, Osaka, Japan; 2 Department of Clinical Epidemiology and Health Economics, School of Public Health, The University of Tokyo, Tokyo, Japan; 3 Translational Research Program, Osaka Medical College, Osaka, Japan; 4 Department of Health Policy and Informatics, Tokyo Medical and Dental University Graduate School of Medicine, Tokyo, Japan

**Keywords:** DIC, Mortality trends, Sepsis, Cancer, Leukemia, Anticoagulants, Descriptive studies

## Abstract

**Introduction:**

Existing evidence on the mortality time trends of patients with disseminated intravascular coagulation (DIC) is limited, and whether the mortality trend or quality of care of DIC patients has improved remains unknown. This study aimed to investigate the temporal trend in mortality, patient outcomes, and treatment preferences of several anticoagulants in Japan.

**Methods:**

This retrospective observational study used the Japanese Diagnosis Procedure Combination inpatient database, which contains data from more than 1200 acute-care hospitals in Japan. We identified all adult patients that were diagnosed with DIC from July 2010 to March 2018 and sorted them into one of five predefined underlying conditions: sepsis, solid cancer, leukemia, trauma, or obstetric. The data collected as general outcomes were the 28-day mortality and major bleeding events. We also evaluated anticoagulant use for DIC treatment.

**Results:**

A total of 325,327 DIC patients were included in this study. Regarding the baseline characteristics, an increase in median age, worsened comorbid conditions, and higher illness severity were observed over time. The underlying conditions for DIC were largely unchanged. Over the study period, the 28-day mortality for overall DIC patients decreased from 41.8% (95% CI 41.2%-42.3%) to 36.1% (95% CI 35.6%-36.6%), which is a 14% decrease over the 8-year period (*P*_trend_ < 0.001). The downward trend in mortality was more evident in patients with sepsis and leukemia (15% and 14% decreases, respectively), whereas no clinically meaningful change in mortality occurred in trauma and obstetrics patients. Over time, major bleeding events modestly increased, and the length of hospital stay decreased. The temporal trend in the treatment preferences of anticoagulants for DIC patients clearly changed over time.

**Conclusions:**

The overall 28-day mortality for DIC patients clearly decreased from 2010 to 2017. The downward trend in mortality might have resulted from the advances made in the fundamental treatment of underlying diseases and from the changes in anti-DIC strategies.

## Introduction

Disseminated intravascular coagulation (DIC) was defined as “*an acquired syndrome characterized by the intravascular activation of coagulation with loss of localization arising from different causes*” by the International Society on Thrombosis and Haemostasis (ISTH) in 2001 ^(1)^. Regarding the relation between DIC presentation and clinical course or patient outcome, ISTH also mentioned the following: “*If sufficiently severe, DIC can produce organ dysfunction*.” Many studies have shown that the mortality of patients with DIC was almost twofold higher than that of patients without DIC in various underlying diseases ^(2), (3), (4), (5)^. Gando et al. ^(6)^ reviewed this complicated syndrome in 2016 and emphasized the necessity for a better understanding of the pathophysiological mechanism of DIC and for the determination of the best clinical management for DIC patients.

Despite its clinical importance, existing evidence regarding the mortality time trend of DIC patients has been limited thus far. Rough estimations have suggested an improvement in the mortality of DIC patients over the past two decades. A nationwide epidemiological survey in Japan showed that the mortality of patients with DIC was as high as 65% in 1992 but decreased to 56% in 1998 ^(6)^. However, the pathophysiological mechanisms of DIC vary in each patient and each population. Therefore, it may be difficult to speculate the downward trend in mortality by comparing several studies involving completely different populations. Murata et al. ^(7), (8)^ used the Japanese national administrative database and found a favorable time trend in DIC mortality from 2012 to 2014. However, in a single-center retrospective cohort study, Singh et al. ^(9)^ reported that the trend in mortality for 154 DIC patients did not change from 2004 to 2010. Taken together, it is still unknown whether the trend in mortality or the quality of care among DIC patients has improved.

To address these knowledge gaps, we investigated the temporal trends in mortality, patient outcomes, and treatment preferences of several anticoagulant therapies from 2010 to 2017 by using a nationwide retrospective dataset of the hospitalized adult DIC population in Japan.

## Materials and Methods

### Design and setting

This study was a retrospective observational study that used routinely collected data. The study was approved by the Institutional Review Board of the University of Tokyo (approval number: 3501-[1] [July 25, 2011]). The board waived the requirement for informed consent because of the anonymous nature of the data and because no information on individual patients, hospitals, or treating physicians was obtained.

We used the Japanese Diagnosis Procedure Combination inpatient database, which includes discharge abstracts and administrative claims data for more than 1200 acute-care hospitals and covers approximately 90% of all tertiary-care emergency hospitals in Japan. The database includes data on age, sex, primary diagnoses, concomitant diagnoses, complication diagnoses, procedures, prescriptions, and discharge status. In this database, the diagnoses are recorded using International Classification of Diseases Tenth Revision (ICD-10) codes and are written in Japanese. Considering that the diagnostic records are linked to a payment system, attending physicians are required to report objective evidence for their diagnosis for the purposes of treatment cost reimbursement. A previous study established the validity of the DIC diagnoses in this database at a diagnostic sensitivity and specificity of 35.8% and 98.2%, respectively ^(10)^. The sensitivity and specificity of treatments or procedures were reported to exceed 90% ^(11)^.

### Study population

We identified all adult patients diagnosed with DIC during hospitalization from July 1, 2010, to March 31, 2018. All hospitalized patients who were diagnosed as having DIC (ICD-10 codes D65, O450, O460, O723, and O081) in the primary, concomitant, or complication diagnoses were included in the study. In other words, DIC diagnoses were based on the physicians’ ICD-10 diagnoses and not on the established diagnostic criteria for DIC.

All patients diagnosed with DIC were identified and sorted into one of five predefined underlying conditions: sepsis, solid cancer, leukemia, trauma, or obstetric. Other miscellaneous diseases were excluded from this analysis. The classification process was performed using a hierarchical system of diagnosis and a procedure to create mutually exclusive groups (see more details in Supplemental Table S1).

We excluded patients who had a suspected diagnosis of DIC and patients who were younger than 18 years of age. We also excluded patients with a diagnosis of DIC who were admitted more than once during the study period (had a second or subsequent record of admission), patients with Child-Pugh class C cirrhosis, and patients with an undetermined category of diseases underlying the DIC.

### Data collection

We collected the following data on baseline patient characteristics: age, sex, Charlson comorbidity index ^(12)^, planned or emergency admission, intensive care unit admission, high care unit admission, surgery with general anesthesia, use of antibiotics, annual hospital volume, underlying conditions of DIC, anticoagulants for DIC, antifibrinolytic for DIC, and blood components for DIC. The anticoagulants for DIC treatment included antithrombin, recombinant human soluble thrombomodulin, serine protease inhibitor (gabexate mesylate or nafamostat mesilate), and heparin (unfractionated heparin ≥ 10,000 units or low molecular weight heparin). Patients in whom an anticoagulant was used one or more times were considered positive for anticoagulant use.

We collected the following data on general outcomes: 28-day mortality, length of hospital stay, and total hospitalization cost. We assessed major bleeding events as adverse events of DIC and anticoagulant therapy by using the ICD-10 codes from the diagnosis of intracranial bleeding (I60-I62 and S064-S066); major gastrointestinal bleeding (I850, I864, K250, K252, K254, K256, K260, K262, K264, K266, K270, K272, K274, K276, K280, K282, K284, K286, K625, and K920-K922); respiratory (J942 and R04), renal/urinary tract (R31), ocular (H313 H356 H431 H450), retroperitoneal (K661), or pericardial bleeding (I312); and bleeding attributed to anemia (D500 and D62). This definition was used previously ^(13)^.

### Statistical analysis

Categorical variables are presented as numbers and percentages. Continuous variables are presented as mean and standard deviation or median and interquartile range. Trend analysis was performed using the nonparametric test, an extension of the Wilcoxon rank test for continuous variables, and the Cochran-Armitage test for binomial proportions across levels of an ordinal variable (e.g., calendar years). We did not adjust for covariates because the study objective was to examine the temporal trends (rather than the causal relationship between calendar years and outcomes). All statistical inferences were performed with a two-sided *p* at the 5% significance level. All analyses were performed using STATA/MP 16.0 software (StataCorp).

## Results

### Study population

**Figure 1.**
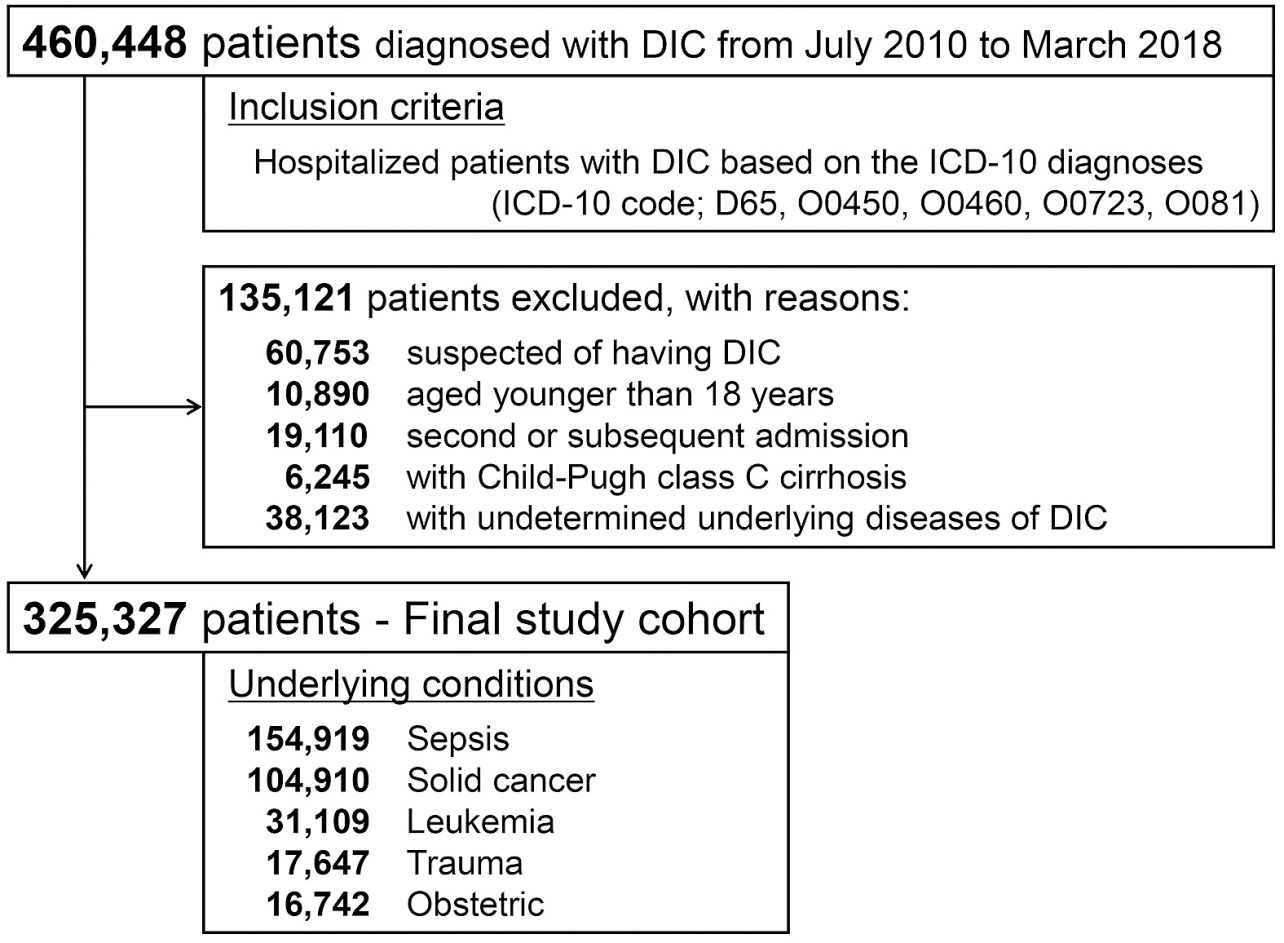
Patient flow diagram. DIC, disseminated intravascular coagulation; ICD, International Classification of Diseases.

After the application of the inclusion and exclusion criteria, a total of 325,327 DIC patients were included in the study (Figure 1). Table 1 shows the baseline patient characteristics by year. From 2010 to 2017, an increase in patient mean age and a decrease in the percentages of males were observed during the study period. The Charlson comorbidity index indicated that the baseline comorbid conditions gradually increased. Illness severity also increased over the study period, as suggested by the proportion of patients arriving via emergency medical services or requiring ICU admission. The most common conditions underlying the DIC were sepsis and solid cancer, and their rates were largely unchanged over the study period.

**Table 1.** Patient Characteristics of Disseminated Intravascular Coagulation in Japan, 2010-2017.

	2010	2011	2012	2013	2014	2015	2016	2017	*P*_trend_^a^
	(n = 30,172)	(n = 42,946)	(n = 45,312)	(n = 43,652)	(n = 42,409)	(n = 39,453)	(n = 42,459)	(n = 38,924)	
Age, years, mean	70 (69-70)	70 (70)	70 (70)	70 (70)	71 (71)	71 (71)	72 (72)	72 (72)	< 0.001
Male sex, %	55 (54-55)	55 (54-55)	54 (54-55)	55 (54-55)	54 (53-54)	54 (53-54)	54 (54-55)	54 (53-54)	< 0.001
Charlson comorbidity index, mean	1.5 (1.4-1.5)	1.4 (1.4-1.5)	1.4 (1.4-1.5)	1.4 (1.4-1.4)	1.4 (1.4-1.4)	1.4 (1.4-1.4)	1.6 (1.6-1.6)	1.6 (1.5-1.6)	< 0.001
Total SOFA score, mean	2.0 (2.0)	2.0 (2.0)	2.0 (2.0)	2.0 (2.0)	2.0 (2.0)	2.0 (2.0)	2.0 (2.0-2.1)	2.1 (2.0-2.1)	< 0.001
Arrived via EMS, %	37 (37-38)	38 (38-39)	40 (39-40)	42 (42-43)	44 (44-45)	46 (45-46)	48 (48-49)	49 (49-50)	< 0.001
ICU admission, %	31 (31-32)	32 (31-32)	36 (35-36)	37 (37-38)	38 (37-38)	40 (39-40)	41 (40-41)	42 (42-43)	< 0.001
Underlying conditions, %									
Sepsis	47 (46-47)	48 (47-48)	46 (45-46)	47 (47-48)	48 (47-48)	49 (48-49)	49 (48-49)	48 (48-49)	<0.001
Solid cancer	33 (33-34)	33 (32-33)	34 (33-34)	32 (32-33)	32 (32-32)	31 (30-31)	32 (31-32)	31 (31-32)	< 0.001
Leukemia	9 (9-10)	10 (9-10)	10 (9-10)	9 (9-10)	10 (9-10)	10 (10)	9 (9-10)	10 (9-10)	0.6302
Trauma	6 (5-6)	5 (5)	5 (5-6)	5 (5-6)	5 (5)	5 (5-6)	6 (5-6)	6 (6)	< 0.001
Obstetric	5 (5)	5 (5)	6 (5-6)	5 (5-6)	5 (5-6)	5 (5)	5 (4-5)	5 (5)	0.1132
Surgery with general anesthesia, %	25 (24-25)	25 (24-25)	27 (27)	26 (26-27)	26 (25-26)	25 (25-26)	24 (24-25)	24 (24-25)	< 0.001
Annual hospital volume, patients per year, mean	74 (74-75)	71 (71-72)	74 (73-74)	74 (73-74)	71 (71-72)	71 (71-72)	69 (68-69)	70 (70-71)	< 0.001

Data are expressed as percent or mean with 95% confidence interval, as indicated. ^a^*P* value for trend test used the nonparametric test for continuous variables or the Cochran-Armitage test for binomial proportions, as appropriate. SOFA, Sequential Organ Failure Assessment; EMS, emergency medical services; ICU, intensive care unit

### Time trends of patient outcomes

**Figure 2.**
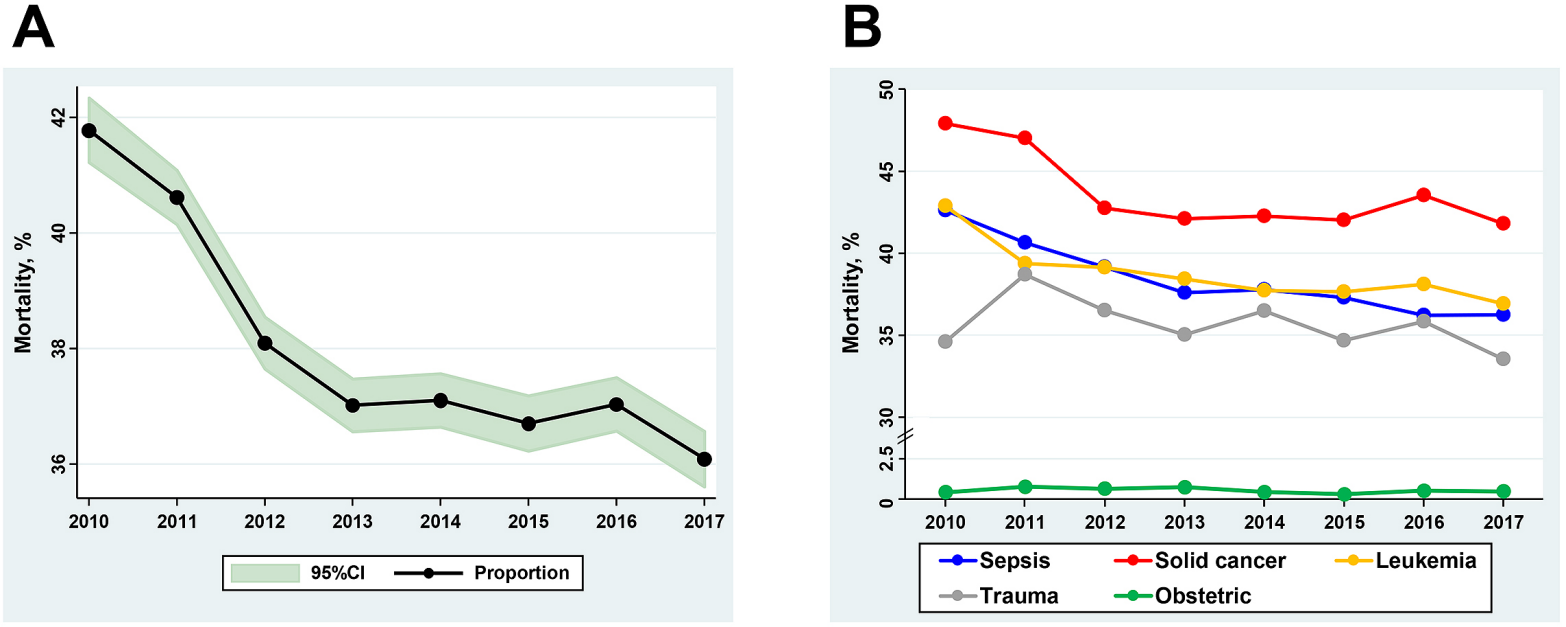
Twenty-eight day mortality for patients with disseminated intravascular coagulation (DIC), 2010-2017. (A) Overall DIC population, and (B) 28-day mortality according to different baseline diseases. Overall mortality in the DIC proportion increased from 2000 to 2017 (14% decrease; *P*_trend_ < 0.001). Similarly, there was a significant decrease in mortality across all different baseline diseases, except for obstetric patients with DIC.

Table 2 shows the overall trends in outcome measures for DIC patients. From 2010 to 2017, the 28-day mortality decreased from 41.8% (95% CI 41.2%-42.3%) to 36.1% (95% CI 35.6%-36.6%), which is a 14% decrease over 8 years (*P*_trend_ < 0.001) (Figure 2A). Major bleeding events among the DIC patients increased over the eight-year period from 8.4% (95% CI 8.1%-8.7%) to 10.4% (95% CI 10.0%-10.7%), which was a 24% increase (*P*_trend_ < 0.001). There was a decrease in the length of hospital stay from 44 days (95% CI 43-45 days) to 40 days (95% CI 39-41 days) (*P*_trend_ < 0.001). The total direct cost per hospitalization increased from ¥2,480,000 (95% CI ¥2,450,000-¥2,510,000) to ¥2,820,000 (95% CI ¥2,790,000-¥2,860,000) (*P*_trend_ < 0.001).

**Table 2.** Outcome Measures in Patients with Disseminated Intravascular Coagulation, 2010-2017.

	2010	2011	2012	2013	2014	2015	2016	2017	*P*_trend_^a^
	(n = 30,172)	(n = 42,946)	(n = 45,312)	(n = 43,652)	(n = 42,409)	(n = 39,453)	(n = 42,459)	(n = 38,924)	
Death within 28 days, %	42 (41-42)	41 (40-41)	38 (38-39)	37 (37)	37 (37-38)	37 (36-37)	37 (37)	36 (36-37)	< 0.001
Major bleeding, %	8 (8-9)	9 (8-9)	9 (8-9)	9 (8-9)	9 (8-9)	8 (8-9)	10 (10-11)	10 (10-11)	< 0.001
Length of stay, days, mean	44 (43-45)	44 (43-45)	44 (43-45)	42 (41-42)	40 (40-41)	40 (39-40)	40 (39-41)	40 (39-41)	< 0.001
Total hospitalization costs, 10,000 yen, mean	248 (245-251)	257 (253-261)	281 (278-284)	278 (276-282)	271 (268-274)	278 (275-281)	278 (275-281)	282 (279-286)	< 0.001

Data are expressed as percent or mean with 95% confidence interval, as indicated. ^a^*P* value for trend test using the nonparametric test for continuous variables or the Cochran-Armitage test for binomial proportions, as appropriate.

Figure 2B and Supplemental Table S2 show the trends in mortality according to the underlying condition for DIC. The downward trend in mortality was more evident in the subpopulations with sepsis and leukemia: 15% decrease (42.6% [95% CI 41.8%-43.4%] to 36.3% [95% CI 35.6%-36.9%]) and 14% decrease (42.9% [95% CI 41.0%-44.7%] to 36.9% [95% CI 35.3%-38.5%]), respectively. However, there was no clinically meaningful change in the 28-day mortality during the study period in the subpopulations of trauma patients and obstetrics patients.

### Time trends of treatment option

**Figure 3.**
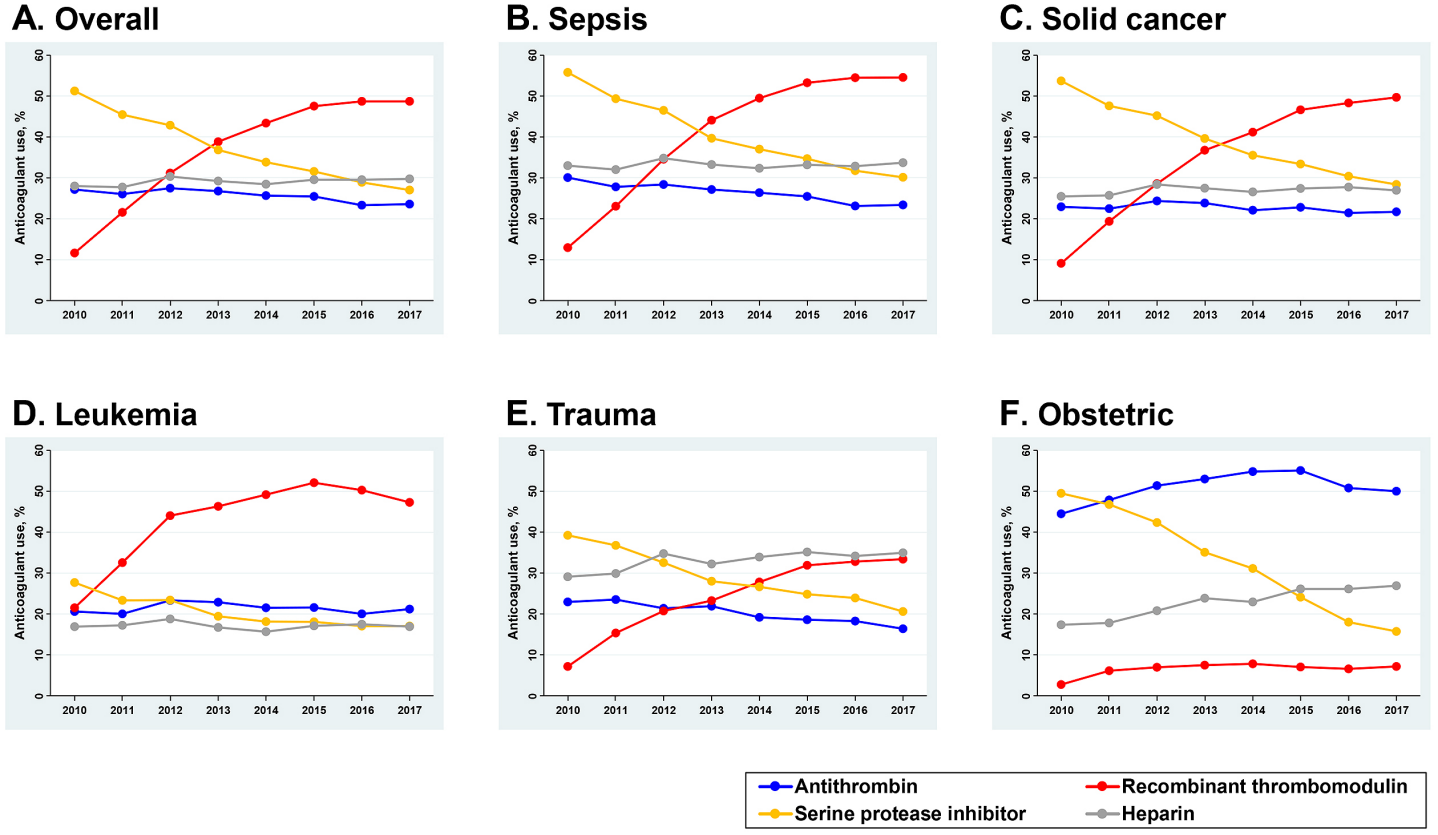
Time trends of anticoagulant uses for patients with disseminated intravascular coagulation, 2011-2017. (A) Overall, (B) sepsis, (C) solid cancer, (D) leukemia, (E) trauma, and (F) obstetric patients.

Figure 3 shows the time trend of the proportion of patients receiving anticoagulants including antithrombin, recombinant thrombomodulin, serine protease inhibitor, and heparin. For the overall population with DIC in Figure 3A, recombinant thrombomodulin administration significantly increased from 11.6% (95% CI 11.2%-12.0%) to 48.7% (95% CI 48.2%-49.2%), which was a 320% increase over the eight years. Alternatively, the use of serine protease inhibitor decreased from 51.2% (95% CI 50.7%-51.8%) to 27.0% (95% CI 26.6%-27.5%), which was a 47% decrease over the same period. The use of antithrombin slightly decreased from 27.1% (95% CI 26.6%-27.6%) to 23.6% (95% CI 23.2%-24.0%), and the use of heparin was approximately unchanged.

Figure 3B, 3C, 3D, 3E, and 3F show the trends of anticoagulant use according to the condition underlying DIC. The upward trend of recombinant thrombomodulin use and the downward trend of serine protease inhibitor were consistently observed across all subpopulations. There were obvious increases in the use of recombinant thrombomodulin for the subpopulations with sepsis and solid cancer: a 320% increase from 12.9% (95% CI 12.4%-13.5%) to 54.6% (95% CI 53.8%-55.3%) and a 450% increase from 9.1% (95% CI 8.5%-9.7%) to 49.7% (95% CI 48.8%-50.6%), respectively. For the subpopulation with leukemia, the baseline use of recombinant thrombomodulin in 2010 was already high at 21.5% (95% CI 20.0%-23.1%) and further increased to 47.3% (95% CI 45.7%-48.9%) in 2017. The preferences of anticoagulant selection in obstetric patients with DIC differed from the other underlying conditions. Antithrombin was mainly used for DIC treatment, and the trend in its use continued to increase during the study period: from 44.5% (95% CI 42.0%-47.0%) in 2010 to 50.0% (95% CI 47.8%-52.3%) in 2017.

## Discussion

### Principal findings

In this study, we analyzed the temporal trends in mortality among DIC patients from 2010 to 2017 by using a nationwide retrospective dataset of 325,327 adult hospitalized patients with DIC in Japan. Over the study period, the 28-day mortality for the overall DIC patients clearly decreased from 41.8% to 36.1%, which was a 14% decrease over the 8 years. This trend in mortality was more evident in the subpopulations with sepsis and leukemia than in other subpopulations, such as trauma patients and obstetrics patients.

### Clinical application of the findings

Several different Clinical Practice Guidelines for DIC have been developed by societies in Britain ^(14)^, Japan ^(15)^, and Italy ^(16)^, along with the harmonized guidance by the ISTH ^(17)^. Although some distinct discrepancies in the appraisal of anticoagulant therapies for DIC exist between these guidelines, the principle concept is consistent: the “*Key to the treatment of DIC is the specific and vigorous treatment of the underlying disorder*” ^(14)^. The present study showed a favorable downward trend in mortality. Even though a promising pathophysiological mechanism of anticoagulant therapy against DIC has been determined, there has been no established evidence of the effectiveness of anti-DIC therapy. Historically, most randomized trials of anticoagulant therapies have failed over the past few decades ^(18), (19), (20), (21), (22), (23)^. However, several previous studies have shown consistent findings for the effectiveness of anticoagulant therapies in sepsis ^(24), (25)^. According to this viewpoint, the downward trend in mortality shown in the present study might be generated not only from the changes in anti-DIC treatment with anticoagulants but also from advances made in the fundamental treatment of the underlying diseases themselves. This clear downward trend in mortality was evident in patients with sepsis and leukemia in this analysis, and both of these diseases have recently been reported to show better mortality trends worldwide ^(26), (27), (28), (29), (30)^. Considering that the present study objective was to examine temporal trends (rather than the causal relationship between calendar years and outcomes), we did not adjust for covariates. Therefore, we cannot answer the clinical question of whether the alterations in the use of anticoagulants in DIC patients are associated with this downward trend in mortality from the present analysis.

Other important outcomes also need to be taken into consideration in the clinical situation. Although the length of hospital stay decreased, the total direct cost per hospitalization tended to increase. The major bleeding events in the DIC patients increased by 24% overall during the eight-year period. As mentioned above, the causal relationship between these time trends in outcome and changes in the preferences of anticoagulant use is unknown. Although previous clinical evidence has suggested a risk of bleeding due to anticoagulant therapy ^(31), (32), (33)^, we must strictly optimize the target populations of these interventions among DIC patients ^(34)^.

### Limitations

This study has several limitations. First, the diagnoses recorded in administrative claims databases are generally less accurate than the diagnoses recorded in planned prospective studies. There could be potential errors in recording diagnoses because of the underestimation or overestimation of the mortality rate over time. Similarly, the misclassification of underlying conditions might have occurred. However, because we used the same hierarchical system to diagnose DIC and classify the underlying diseases, the main findings in this study, that is, the mortality time trend and treatment options, may not have been influenced by these limitations. In fact, the observed trends revealed consistent trends (e.g., the consistent decrease in mortality and changes in anticoagulant use) rather than year-by-year fluctuations. Second, our diagnoses of DIC were based on ICD-10 diagnoses recorded by attending physicians and may not have been strictly based on the established diagnostic criteria. Given that the applied diagnostic criteria for DIC may differ according to the underlying conditions, the baseline characteristics of the targeted DIC population with each underlying condition might be different from one underlying condition to another. Third, the cause of death could not be obtained from the dataset used. Although we assessed the mortality trends of DIC patients in this study, whether the mortality events were directly caused by DIC is unclear. Finally, we could not obtain the results of any coagulation tests, established disease scoring systems, or vital signs. Thus, we did not assess the baseline disease severity of each subpopulation directly.

### Conclusions

This nationwide retrospective observational study of 325,327 adult DIC patients shows that the overall 28-day mortality for DIC patients clearly decreased from 41.8% to 36.1% over the 8-year study period. The downward trend in mortality was more evident in the subpopulations with sepsis and leukemia and might have been generated from the advances made in the fundamental treatment of the underlying diseases and from the changes in anti-DIC strategies.

## Article Information

### Conflicts of Interest

None

### Sources of Funding

This work was supported by the Ministry of Education, Culture, Sports, Science and Technology, Japan [grant number 19H03759].

### Author Contributions

KY and HO contributed equally to this work. KY and HO conceived and designed this study; contributed to acquisition, analysis, and interpretation of the data; and were responsible for drafting, editing, and submission of the manuscript. KT contributed to the design of the study, the interpretation of the data, and drafting of the manuscript. HM, KF, and HY had a significant influence on the interpretation of the data and critical appraisal of the manuscript. All of the authors contributed to the acquisition of data, reviewed, discussed, and approved the final manuscript.

### Approval by Institutional Review Board (IRB)

The study was approved by the Institutional Review Board of the University of Tokyo [approval number: 3501-(1) (July 25, 2011)].

## Supplement

Supplemental Table
