# Supplementary material for: Time Trends of the Outcomes and Treatment Options for Disseminated Intravascular Coagulation: A Nationwide Observational Study in Japan
Source: JMA J. 2020 Sep 23;3(4):313–20. doi: 10.31662/jmaj.2020-0013 (PMC7677444; doi:10.31662/jmaj.2020-0013)
Supplement: Supplementary file 1 — Supplemental Table [file 2433-3298-3-4-0313-s001.pdf]

Table S1. Diagnosis and procedure code-based criteria to define underlying condition for disseminated intravascular coagulation

Group	Code type	Code	Related criteria
Sepsis	ICD-10 codes in the primary, concomitant or complication diagnoses	A02 A04 A05 A08 A09 A15-A19 A27 A28 A31 A32 A35 A37 A39 A40-A43 A46 A48 A49 A52-A54 A69 B35 B36 B374-B379 B44-B46 B48 B49 G00-G09 I30 I33 I80 J01-J06 J13-J18 J20-J22 J440 J441 J47 J69 J85 J86 K35-K37 K57 K61 K630 K631 K65 K67 K750 K751 K80 K81 K830 K918 L00 L03 L04 L08 L88 M00 M86 N10-N12 N151 N159 N160 N30 N34 N390 N41 N70-N73 N76 N77 T814 T826 T827 T835 T845-T847 T857	Absence of codes to qualify for any of 5 groups
Solid cancer	ICD-10 codes in the primary, concomitant or complication diagnoses	C00-C80 C97 D37-D48	Absence of codes for Obstetric, or Leukemia
Leukemia	ICD-10 codes in the primary, concomitant or complication diagnoses	C81-C96	Absence of codes for Obstetric
Trauma	ICD-10 codes in the primary, concomitant or complication diagnoses	S00-T14	Absence of codes for Obstetric, Leukemia, Solid cancer, or Miscellaneous
Obstetric	ICD-10 codes in the primary diagnoses	O00-O99	
	Japanese DPC code form 1	Existence of the pregnancy during hospitalization	
Miscellaneous	ICD-10 codes in the primary diagnoses	D180 G210 I46 I490 I70 I72 I74 K550 M30 M310 M312-M319 M6289 N280 T20-T32 T630 T67 T68	Absence of codes for Obstetric, Leukemia, or Solid cancer

ICD-10 indicates International Classification of Diseases, Tenth Revision; DPC, Diagnosis Procedure Combination.

Table S2. Twenty-eight day mortality of patients with disseminated intravascular coagulation according to the underlining conditions, 2010-2017

	2010 (n=30,172)	2011 (n=42,946)	2012 (n=45,312)	2013 (n=43,652)	2014 (n=42,409)	2015 (n=39,453)	2016 (n=42,459)	2017 (n=38,924)	P_{trend}[a]
Overall, %	42 (41-42)	41 (40-41)	38 (38-39)	37 (37-37)	37 (37-38)	37 (36-37)	37 (37-37)	36 (36-37)	<0.001
Sepsis, %	43 (42-43)	41 (40-41)	39 (38-40)	38 (37-38)	38 (37-38)	37 (37-38)	36 (36-37)	36 (36-37)	<0.001
Solid cancer, %	48 (47-49)	47 (46-48)	43 (42-44)	42 (41-43)	42 (41-43)	42 (41-43)	44 (43-44)	42 (41-43)	<0.001
Leukemia, %	43 (41-45)	40 (38-41)	40 (38-41)	38 (37-40)	38 (36-40)	38 (36-40)	38 (37-40)	37 (35-38)	<0.001
Trauma, %	35 (32-37)	39 (37-41)	37 (35-38)	35 (33-37)	36 (34-39)	35 (33-37)	36 (34-38)	34 (32-36)	0.029
Obstetric, %	4 (2-9)	8 (5-13)	7 (4-11)	8 (5-12)	5 (2-8)	3 (1-7)	6 (3-10)	5 (2-9)	0.264

Data are expressed as percent or mean with 95% confidence interval, as indicated.

[a]*P* value for trend test using the Cochran-Armitage test for binomial proportions.
